# Physicochemical properties and formulation development of a novel compound inhibiting *Staphylococcus aureus* biofilm formation

**DOI:** 10.1371/journal.pone.0246408

**Published:** 2021-02-08

**Authors:** Nan Wang, Feng Qi, Haqing Yu, Bryan D. Yestrepsky, Scott D. Larsen, Honglan Shi, Juan Ji, David W. Anderson, Hao Li, Hongmin Sun

**Affiliations:** 1 Department of Mechanical and Aerospace Engineering, University of Missouri, Columbia, Missouri, United States of America; 2 Division of Cardiovascular Medicine, Department of Medicine, University of Missouri, Columbia, Missouri, United States of America; 3 Vahlteich Medicinal Chemistry Core, College of Pharmacy, University of Michigan, Ann Arbor, Michigan, United States of America; 4 Department of Chemistry, Missouri University of Science and Technology, Rolla, Missouri, United States of America; 5 Department of Biochemistry, University of Missouri, Columbia, Missouri, United States of America; 6 Ivogen Inc. (Subsidiary of Nanova, Inc.), Columbia, Missouri, United States of America; Cairo University, EGYPT

## Abstract

The emergence of antibiotic resistance over the past several decades has given urgency to new antibacterial strategies that apply less selective pressure. A new class of anti-virulence compounds were developed that are active against methicillin-resistant *Staphylococcus aureus* (MRSA), by inhibiting bacterial virulence without hindering their growth to reduce the selective pressure for resistance development. One of the compounds CCG-211790 has demonstrated potent anti-biofilm activity against MRSA. This new class of anti-virulence compounds inhibited the gene expression of virulence factors involved in biofilm formation and disrupted the biofilm structures. In this study, the physicochemical properties of CCG-211790, including morphology, solubility in pure water or in water containing sodium dodecyl sulfate, solubility in organic solvents, and stability with respect to pH were investigated for the first time. Furthermore, a topical formulation was developed to enhance the therapeutic potential of the compound. The formulation demonstrated acceptable properties for drug release, viscosity, pH, cosmetic elegance and stability of over nine months.

## Introduction

The rapid evolution and spread of antibiotic-resistant bacteria over the past several decades have made antibiotic resistance a worldwide medical threat to humans [1, 2]. Conventional antibiotics exert strong natural selection for drug-resistant bacteria by killing or inhibiting drug-sensitive competitors [3]. The increasing occurrence of antibiotic-resistant strains calls for new antibacterial strategies that apply less selective pressure [4]. Antibiotic-resistance has appeared in most common human pathogens. Among them, methicillin-resistance in *Staphylococcus aureus* (MRSA) is approaching epidemic level [5, 6]. *Staphylococcus aureus* (*S*. *aureus*) is one of the most common human pathogens, causing skin, soft tissue, respiratory, bone, joint and endovascular infections, including life-threating bacteremia, endocarditis, sepsis and toxic shock syndrome [7]. Novel antimicrobial agents that can replace or complement conventional antibiotics could significantly enrich the therapeutic arsenal against antibiotic resistant bacterial infections.

The novel anti-virulence approach of inhibiting pathogen virulence or disarming their pathogenic weapons, while minimizing the selection pressure for resistance, is gaining recognition as a viable alternative to traditional antibiotic treatment [8–11]. With the number of anti-virulence studies growing exponentially in recent years, progress is being made toward new approaches [12, 13]. However, most of the studies for anti-virulence molecules are still in the preclinical stages. Only very recently, this anti-virulence paradigm started to bear fruit. For example, Zinplava™ (Bezlotoxumab; Merck, Kenilworth, NJ, USA) was approved in 2016 as one of the first anti-virulence drugs that can neutralize the toxin B of *Clostridium difficile* to reduce recurrence and lower colitis and diarrhea, illustrating the tremendous potential of this approach for solving the urgent antibiotic resistance problem. Other anti-virulence/anti-toxin approaches have also advanced compounds into clinical development [14, 15].

A class of quinazolin-4-one derivatives were identified through high throughput screening that inhibited the expression of a key *Streptococcus pyogenes*, or group A streptococcus (GAS), virulence factor streptokinase, with the lead compound subsequently demonstrating *in vivo* efficacy against GAS infection [16]. More studies demonstrated that analogs of the lead compound also inhibited the biofilm formation of *S*. *aureus* [17]. These compounds block the production of key virulence factors, especially those involved in biofilm formation. Biofilm is a virulence mechanism that allows bacteria to colonize tissues and provide a physical barrier to shield the bacteria from the body’s disease fighting white blood cells and antibodies. Biofilm consists of complex structures of bacteria enveloped in extracellular polymeric matrix with polysaccharides and water, together with excreted cellular products such as extracellular DNA (eDNA), which stabilizes biofilm structures and contributes to antimicrobial resistance [18–23]. Biofilm formation also contributes to antibiotic resistance by providing a physical barrier to antibiotics [24, 25]. Infections caused by biofilm-associated pathogens are difficult to eradicate because the biofilm structure, extracellular matrix, and inter-bacterial signaling of microbial biofilm communities prevent antibiotic penetration, limit nutrition, promote bacterial persistence and contribute to the antibiotic resistance of biofilm bacteria [18, 22, 23, 26–29]. Therefore, the production of biofilm-associated virulence factors makes bacteria harder to treat and can transform infections from acute to chronic stage and lead to serious complications [27, 30]. *S*. *aureus* is one of the most frequent causes of biofilm-associated clinical infections [23, 28]. *S*. *aureus* is also a leading pathogen that may cause a variety of diseases ranging from moderate to severe skin and soft tissue infections to very serious diseases such as endocarditis, osteomyelitis, septic shock, toxic shock syndrome, or necrotizing pneumonia [23, 28, 31]. Agents that decrease the protective biofilm will improve the effectiveness of treatment of recalcitrant infections [21, 23, 30].

The current study reported a new anti-virulence compound CCG-211790 against *S*. *aureus*. The physicochemical properties of CCG-211790, including morphology, solubility in pure water or in water containing sodium dodecyl sulfate (SDS), solubility in organic solvents, and stability with respect to pH were investigated for the first time. A topical formulation of this novel class of anti-virulence compounds was developed and characterized. The new formulation with anti-virulence compounds has potential to be developed into therapies to treat skin and soft tissue infections (SSTI), to help overcome increasing incidence of antibiotic resistance and formation of biofilm often leading to treatment failures with conventional antibiotics [24, 25].

## Materials and methods

### Materials

CCG-211790 was designed in the Vahlteich Medicinal Chemistry Core (VMCC) laboratory at the University of Michigan. The compound was re-synthesized for the present studies in gram scale by Active Biochem LTD (Kowloon, Hong Kong, China) by a proprietary route. Described below is the lab scale synthesis developed by the VMCC. Ethanol was purchased from Decon Laboratories Inc. (Detroit, MI, USA). SDS, glycerin, isopropyl alcohol, Dimethylacetamide (DMA), dimethylformamide (DMF) and acetonitrile were purchased from Fisher Scientific (Pittsburgh, PA, USA). Tween^®^80 (polyoxyethylene-20-sorbitan monooleate) and dimethyl sulfoxide (DMSO) were purchased from Sigma-Aldrich (St. Louis, MO, USA). Propylene glycol was purchased from MP Biomedicals, LLC (Solon, OH, USA). Phosphate buffered saline (10 mM) was purchased from Life Technologies Corp. (Carlsbad, CA, USA). Deionized water was supplied from a reverse osmosis filtration system, Colligan Water (Rosemont, IL, USA) or prepared from an ELIX-3 water purification system (Millipore, Billerica, MA, USA) for HPLC analysis. All the chemicals above were of analytical or high-performance liquid chromatography (HPLC) grade. Liquid coconut oil, peanut oil, olive oil, sunflower oil, sesame oil, canola oil, safflower oil and oleic acid were purchased from Piping Rock Health Products, LLC (Ronkonkoma, NY, USA) and were used as received. Other materials in analytical grade were obtained from Sigma-Aldrich (St. Louis, MO, USA).

### Bacterial strains and culture conditions

Methicillin-resistant *Staphylococcus aureus* (MRSA) USA300 strain NRS384 (Strain USA300-0114, NR-46070), was provided by the Network on Antimicrobial Resistance in *Staphylococcus aureus* (NARSA) for distribution by BEI Resources, NIAID, NIH. The NRS384 strain was grown in Todd-Hewitt broth containing 0.2% yeast extract (THY) (Difco, Detroit, MI, USA). The medium for growth of static biofilms was THY with 0.5% glucose. All bacterial cultures were incubated at 37°C.

### Synthesis of CCG-211790

**Fig 1:**
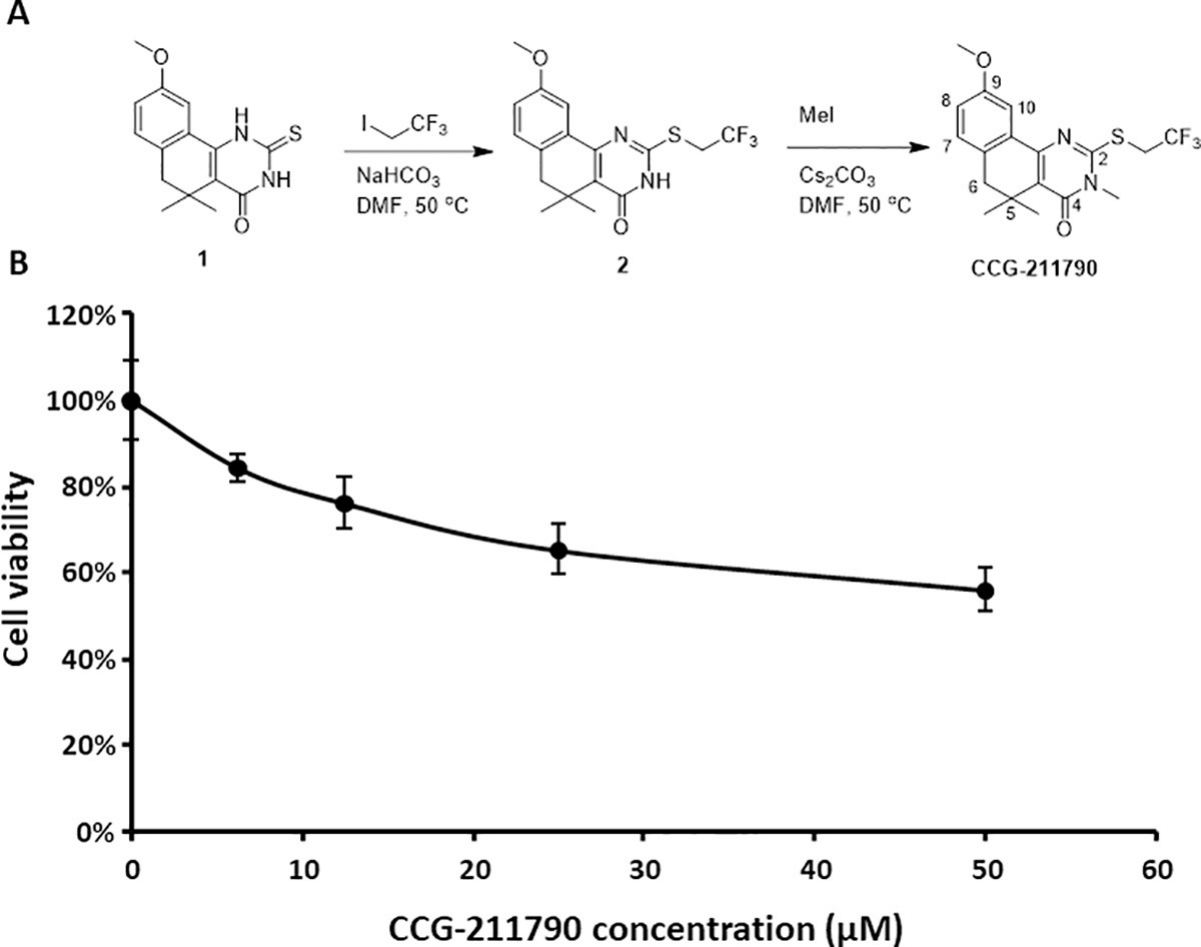
Synthesis and cytotoxicity of CCG-211790 A) Synthesis of CCG-211790 B) Mammalian cell viability in the presence of CCG-211790 at different concentrations normalized to the value for DMSO treated samples which was defined as 100%. The data is presented as mean ± standard deviation for a total of 4 samples (pooled from 2 independent experiments in duplicates).

CCG-211790 was synthesized as in Fig 1A using the following steps:

#### 9-Methoxy-5,5-dimethyl-2-((2,2,2-trifluoroethyl)thio)-5,6-dihydrobenzo[h]quinazolin-4(3H)-one (2)

Intermediate **1** [32] (175 mg, 0.607 mmol) was combined with 1,1,1-trifluoro-2-iodoethane (150 μL, 1.52 mmol) and sodium bicarbonate (1.5 equiv) in DMF (3 mL) and warmed to 50°C. The reaction was allowed to stir for 6 h at 50°C, then was diluted with water (10 mL) and extracted twice with ethyl acetate (2 × 10 mL). The organic extract was washed with water (3 × mL) and brine (1 × 10 mL), then was dried over anhydrous MgSO_4_, vacuum filtered, and concentrated *in vacuo* to give a yellow solid that was used without further purification (208 mg, 93% yield). ^1^H NMR (400 M*Hz*, Chloroform-*d*) δ 11.68 (s, 1H), 7.65 (d, *J* = 2.7 *Hz*, 1H), 7.11 (d, *J* = 8.2 *Hz*, 1H), 6.94 (dd, *J* = 8.2, 2.7 *Hz*, 1H), 4.10 (q, *J* = 9.6 *Hz*, 2H), 3.85 (s, 3H), 2.74 (s, 2H), 1.40 (s, 6H).

#### 9-Methoxy-3,5,5-trimethyl-2-((2,2,2-trifluoroethyl)thio)-5,6-dihydrobenzo[h]quinazolin-4(3H)-one (CCG-211790)

**2** (54 mg, 0.146 mmol) was dissolved in DMF (1 mL), to which methyl iodide (23 mg, 0.16 mmol) and cesium carbonate (1.1 equiv) were added. The reaction was heated to 50°C and allowed to stir for 16 hours, then diluted with H_2_O (10 mL) and extracted 3 × 10 mL with EtOAc. The organic layer was washed with water (10 mL) and brine (10 mL), then separated, dried over anhydrous MgSO_4_, vacuum filtered, and concentrated. Flash chromatography isolated only the N-alkylated isomer (32 mg, 57% yield). ^1^H NMR (600 MHz, CDCl_3_): δ 7.64 (d, *J* = 2.8 Hz, 1H), 7.12 (d, *J* = 8.1 Hz, 1H), 6.95 (dd, *J* = 8.3, 2.9 Hz, 1H), 4.20 (q, *J* = 9.7 Hz, 2H), 3.87 (s, 3H), 3.57 (s, 3H), 2.75 (s, 2H), 1.39 (d, *J* = 3.5 Hz, 6H). ^13^C NMR (151 MHz, CDCl_3_): δ 161.55, 158.61, 155.64, 151.76, 132.75, 129.45, 124.80 (q, *J* = 276.5 Hz), 122.22, 122.05, 116.88, 109.62, 55.19, 43.81, 33.81, 33.22 (q, *J* = 34.2 Hz), 30.30, 25.84. ESI^+^ MS *m/z* = 385.0 (M + H^+^) 407.0 (M + Na^+^). Reverse phase HPLC purity >95%.

### Biofilm assay

The biofilm assay was performed using 96-well polystyrene flat-bottom microtiter plate as described previously [17]. Overnight cultures of *S*. *aureus* were diluted 1:200 with fresh THY medium containing 0.5% glucose. Aliquots of 200 μL bacteria culture was incubated in wells of 96-well microtiter plates with different concentration of CCG-211790 (0.39, 0.78, 1.56, 3.13, 6.25, 12.5, 25, 50 μM) or control DMSO in triplicate at 37°C overnight. Non-adherent bacteria were washed away by PBS three times. Biofilms attached to microtiter plates were stained by crystal violet solution (Sigma-Aldrich, St. Louis, MO, USA) for 15 min. Excess stain was removed by washing with PBS. The crystal violet attached to biofilm samples was dissolved with ethanol. The absorbance at 595 nm was measured using Spectrostar Nano spectrophotometer (BMG Labtech, Ortenberg, Germany) as the value of biofilm formation. Percentage inhibition of sample treated with CCG-211790 was calculated against the mean of samples treated with DMSO. Experiments were repeated three times to obtain the mean and standard deviation of biofilm formation under each treatment. IC_50_ (the half maximal inhibitory concentration) was estimated.

### Microsomal stability

Microsomal stability assay was performed as previously described [32]. CCG-211790 (100 mM stock solution in DMSO) was diluted 1000-fold with phosphate-buffered saline (PBS) containing up to 10% MeOH as a co-solvent. Compound (100 μM) was added to 366 μL of 100 mM phosphate buffer containing 3.3 mM MgCl_2_ and 10 μL of 20 mg/mL mouse liver microsomal extract (XenoTech, Lenexa, KS, USA). Enzymatic oxidation was initiated by adding 15 μL of 16.7 mg/mL NADPH in 100 mM phosphate buffer containing 3.3 mM MgCl_2_. The reactions were carried out at 37°C for 60 min. Aliquots of the reaction mixture (30 μL) were sampled and the reaction was stopped by adding 90 μL cold acetonitrile containing an internal standard compound at each time point (0, 1, 3, 5, 10, 30, and 60 min). The quenched sample mixtures were centrifuged at 16,000 g for 10 min. The supernatant was then analyzed via an LC–MS/MS system equipped with a reverse-phase column.

### Mammalian cell cytotoxicity assay

Cell cytotoxicity assay was performed by following previous protocols with minor revision [17]. TZM-bL cells which is a HeLa cell derivative (a generous gift from Dr. Xiao Heng, University of Missouri) (100 μL, 5,000 cells per well) were grown in Dulbecco’s modified Eagle’s medium (DMEM) (Life Technologies, Grand Island, NY, USA) with 10% fetal bovine serum for 24 h at 37°C in 5% CO_2_ in duplicates. Supernatant was replaced with 100 μL fresh medium with different concentrations of CCG-211790 to culture the cells for 24 h at 37°C in 5% CO_2._ DMSO was used as the vehicle control. Ten μL of Cell Counting Kit-8 (CCK-8) solution (APExBIO, Houston, TX, USA) was added to each well of the plate. After 2 h, the plate was read at 450 nm by using a BioTek spectrophotometer (BioTek, Winooski, VT, USA). One hundred percent viability was set at the absorbance of the cells treated with only the vehicle (DMSO). The assay was performed twice in duplicates to obtain the mean and standard deviation of cell viability. The cell viability (survival rate) was calculated with the equation: Survival rate (%) = [(As—Ab) / (Ac—Ab)] x 100, As = Absorbance of tested compound (Absorbance of well containing cells, culture medium, CCK-8 and compound), Ab = Absorbance of blank (Absorbance of well containing culture medium and CCK-8), Ac = Absorbance of control (Absorbance of well containing cell, DMSO and CCK-8).

### Scanning Electron Microscopy (SEM)

Morphology of CCG-211790 bulk powder was examined using a FEI Quanta 600 FEG Environmental Scanning Electron Microscope (FEI Company, Hillsboro, OR, USA). CCG-211790 bulk powder was used as received. The sample was fixed on an aluminum SEM stage with a conductive double-sided carbon tape and sputter-coated with 5 nm of platinum prior to measurement in order to increase sample conductivity. SEM measurements were carried out at an accelerating voltage of 5 kV and a work distance of 8–9 mm.

### Ultraviolet-visible spectrophotometry (UV-Vis)

The concentration of CCG-211790 in organic solvents was carried out on a LabTech BlueStar plus UV-Vis spectrophotometer (Hopkinton, MA, USA) using dimethylformamide (DMF) as a reference solvent. UV-Vis absorption spectra were recorded in a wavelength range from 200–500 nm with samples placed in a quartz cuvette. CCG-211790 concentrations in organic solvents were determined based on the absorption of CCG-211790 at characteristic wavelength of 314 nm. Calibration curves were established giving a correlation coefficient more than 0.999.

### High-Performance Liquid Chromatography (HPLC)

The concentration of CCG-211790 in deionized water or aqueous solutions was determined using an Agilent series 1100 HPLC system (Agilent Technologies, Santa Clara, CA, USA) equipped with diode array detector. Sample separation was carried out with a Kinetex C18 column (75 mm × 3 mm, particle size 2.6 μm, Phenomenex Inc., Torrance, CA, USA). The mobile phase of acetonitrile-water (50:50, *v/v*) was used for chromatographic separation. The flow rate was set to 0.5 mL/min, column temperature to 35°C. The absorption was measured at wavelength 255 nm. The injection volume was 30 μL. The retention time of CCG-211790 was 4.9 min. The method detection limit was 0.02 mg/L. Calibration curves were established giving a correlation coefficient >0.9900.

### Solubility in water

To determine the solubility of CCG-211790 in water [33], an excess amount of CCG-211790 powder was added into water. After incubation for 20 h at room temperature on a shaking platform, it still showed visible milky turbidity. The sample aliquots (0.5 mL each) were centrifuged at 20,000 ×g for 5 min with Amicon Ultra-0.5 Centrifugal Filter Units (MWCO 3000 Da, Millipore Corp., Billerica, MA, USA). Solutions passing through the filter were diluted 2-fold with acetonitrile and CCG-211790 concentration in water was determined by HPLC analysis. The experiment was run in triplicate with mean values and standard deviations being reported.

### Solubility in water containing Sodium Dodecyl Sulfate (SDS)

The procedure to determine solubility in water with presence of SDS is based on the Paddle Method as described in the U.S. Pharmacopeia <711> on the *in vitro* dissolution tests with minor revision. To determine the saturation solubility in water containing SDS, CCG-211790 bulk powder was added in excess (around 5 mg of CCG-211790) to 10 mL of SDS solutions with various concentrations (0.5, 1.0 or 2.0% *w/v*). After incubation for 24–48 h in a shaking incubator at 37°C and 200 rpm to reach saturation status, aliquot (2 mL) of the incubated sample was filtered through a 0.22 μm Millipore filter (Millipore Corp., Billerica, MA, USA). Solutions passing through the filter were collected (0.5 mL) and were diluted 2-fold with acetonitrile. CCG-211790 concentration in SDS solutions was determined by HPLC analysis.

### Solubility in organic solvents

The solubility of CCG-211790 in organic solvents were also determined [33]. CCG-211790 bulk powder was added in excess to 0.1–1 mL of an organic solvent. After ultrasonication for 30 min and incubation for 24 h at room temperature on a shaking platform to reach the saturation status, sample aliquots (0.5 mL) were centrifuged at 10,400 ×g for 10 min, and 10 μL of supernatant was diluted 500–50,000-fold with dimethylformamide (DMF). The saturation solubility of CCG-211790 in various organic solvents was determined via ultraviolet-visible spectrophotometry (UV-Vis) with DMF as the reference solvent.

### Effect of pH on CCG-211790 degradation

Effect of pH on CCG-211790 degradation was investigated at indicated pH (1–10) over a period of three months [34, 35]. Briefly, pH 1, 2 or 10 were achieved by dissolving CCG-211790 bulk powder in a mixture of acetonitrile and water (25:75, *v/v*) with the pH being adjusted with hydrochloride acid solution (12 mole/L) or sodium hydroxide solution (15 mole/L). Similarly, pH 3–9 were achieved by dissolving CCG-211790 powder in a mixture of acetonitrile and PBS (25:75, *v/v*) with the pH also being adjusted with the hydrochloride acid solution or the sodium hydroxide solution. All samples were incubated at 37°C. CCG-211790 concentrations in the mixture solutions were determined via HPLC analysis at predetermined time points (0, 2, 4 and 12 weeks).

### Preparation of cream formulation of CCG-211790

The following protocol was used for preparing 100g CCG-211790 (0.3%, 1%, 3% *w/v*) cream:

Water (33.1%, 32.4%, 30.4% *w/v*), citric acid (0.07% *w/v*), and sodium citrate (0.23% *w/v*) were stirred at room temperature until completely dissolved. Xanthan gum (0.2% *w/v*) was gently added into the solution under vigorously stirring (400 rpm), then stirred at 500 rpm until the gum completely dissolving to be used as aqueous phase (phase A). Isopropyl myristate (IPM, 10.5% *w/v*), mineral oil (42.2% *w/v*), Stearyl alcohol (3.6% *w/v*), cetyl alcohol (3.6% *w/v*), BRIJ® 58 (6.2% *w/v*) and CCG-211790 (0.3%, 1%, 3% *w/v*) were stirred into the oil mixture (phase B). Phase A and phase B were maintained in water bath (70–80°C) for 5–6 min under stirring at 500 rpm. CCG-211790 was added into phase B after maintaining phase B in the water bath for 3 min and stirred at 500 rpm for another 3 min. Phase B solution was added into phase A and immediately homogenized with a high-speed homogenizer (T18, IKA) at 15,000 rpm for 3 min to prepare emulsion. The obtained emulsion was stirred and cooled down to room temperature using a homogenizer (SciLogex OS40-S, SCILOGEX, LLC, Rocky Hill, CT, USA) at 100 rpm for 10 min and then 50 rpm for another 10 min. The cream was ready for use after cooling to room temperature.

### Drug release profile (Franz Chamber Assay)

The Franz Chamber Protocol was used for evaluating drug release using published methods [36, 37] as follows: 1) Prepare receiving medium (2% SDS in Ethanol/Water (40/60)), and degas the medium by ultrasound bath; 2) Turn on the machine and adjust the temperature at 32°C; 3) Saturate the membrane (Polycarbonate (0.4 μm)) in receiving medium for 30 min; 4) Apply membrane on the dosage wafer, and apply spatula to spread out cream on the membrane (200 mg for 0.3% and 1.0%, 100 mg for 3.0%); 5) Add medium into receptor and place the wafer and membrane on the receptor without bubbles; 6) Use clamp to fix the donor and receptor; 7) Seal the donor chamber using sealing film; 8) Set the stirring speed at 600 rpm, and start the machine; 9) Stop stirring 30 seconds before sampling, take 300 μL medium at 0.5 h, 1 h, 2 h, 3 h, 4 h and 6 h, and refill with fresh medium.

### Quality target product profile of the CCG-211790 cream

The oil phase solubilizes the active agent, CCG-211790, which is emulsified with water, surfactants, and thickening agents to provide the desired viscosity and stability. The emulsion has the acceptable properties for drug release (Franz Chamber Analysis), viscosity, pH, cosmetic elegance (no smell, non-greasy, spreadability, quick drying, and non-irritating) and stability with over nine months testing completed.

### Statistic

Statistical analysis of the results was performed using a two-sided Student’s t-test. Dose Response fitting function in Origin was used to calculate the IC_50_. The IC_50_ was calculated by plotting the inhibition percentage (*y*) versus logarithm of dose (*x*), and fitting the data using the equation $y=A_{1}+\frac{A_{2}-A_{1}}{1+10^{(Logx_{0}-x)p}}$. IC_50_ was calculated as $10^{logx_{0}}$. *A*_*1*_ stands for bottom asymptote, *A*_*2*_ stands for top asymptote and *p* is the hill slope (Origin 7.0, OriginLab Corporation, Northampton, MA, USA).

## Results

### Generation of CCG-211790 as an inhibitor of *Staphylococcus aureus* biofilm formation

CCG-211790 was designed and synthesized as part of a structure-relationship analysis (SAR) in developing anti-virulence compounds against *S*. *aureus* biofilm formation (Fig 1A), following the discovery of lead compounds CCG-203592 and CCG-205363 [17, 32]. In the larger SAR program that was described previously [32], 87 analogs of the lead compounds were identified with > 20% inhibition of *S*. *aureus* biofilm at 100 or 50 μM [38]. CCG-211790 was selected for further analysis of its therapeutic potential because of its potent *S*. *aureus* anti-biofilm activity, and its relatively high stability to incubation with mouse liver microsomes.

The effect of CCG-211790 on biofilm formation was tested with *S*. *aureus* NRS384 strain, which is widely used for studying MRSA infections. NRS384 was treated with different concentrations of CCG-211790, and the IC_50_ of the inhibition of biofilm formation was calculated. CCG-211790 inhibited biofilm formation with IC_50_ = 1.28±0.45 μM, which is more potent than the previous lead compounds CCG-203592 and CCG-205363 [17]. The half-life of CCG-211790 in mouse liver microsomes (t_1/2_) is 19.6 min while CCG-203592 (t_1/2_ = 0.8 min) and CCG-205363 (t_1/2_ = 0.6 min) have lower half-life [32].

Stability to mouse liver microsomes is one predictor of half-life *in vivo* in mice. CCG-211790 was designed to be stable to oxidative metabolism of the S-substituent. A metabolic identification study of an early lead analog, described in Yestrepsky et al. [32], indicated that the S-substituent is a primary site of metabolism by CYP enzymes, therefore the terminus of the S-ethyl group with fluorine atoms, which are known to be resistant to oxidative metabolism, was blocked [39]. As a result, CCG-211790 demonstrated a metabolic stability superior to all of the structurally-related compounds evaluated in that earlier work [32]. It also demonstrated more broad-spectrum and potent anti-virulence activity against *Streptococcus pyogenes* (manuscript in preparation). It was thus selected as a candidate for further characterization of its potential to become a therapeutic agent.

Cytotoxicity of CCG-211790 to mammalian cells was also tested on TZM-bL cells which is a HeLa cell derivative. The cytotoxicity of CCG-211790 at concentrations of 6.25, 12.5, 25, and 50 μM was measured by the colorimetric CCK8 viability assay and compared to cell viability when treated with DMSO control (Fig 1B). At 50 μM, the CCG-211790 displayed cytotoxic activity on TZM-bL cells and the cell survival rate was 56.1±5.3%, which is about 39-fold of the anti-biofilm IC_50_ of the compound, indicating that CCG-211790 has a wide selectivity window. Lower concentrations of CCG-211790 have little toxicity against human cells but have significant anti-biofilm activity.

### Morphology

**Fig 2:**
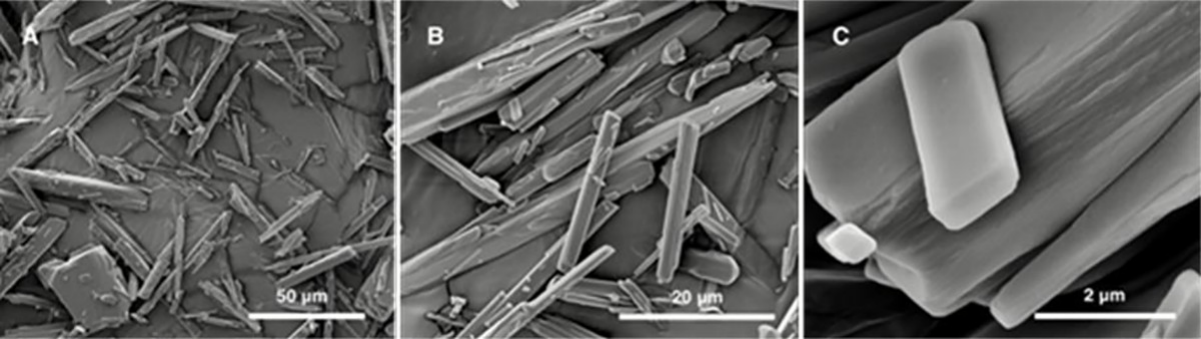
SEM images of CCG-211790 bulk powder after sputter-coating of 5 nm of platinum.

The SEM images of CCG-211790 bulk powder, as seen in Fig 2, showed that CCG-211790 bulk powder consisted of either medium rod-like particles with length in up to twenty micrometers and diameter in a few micrometers, or large, thick particles with length over fifty micrometers and diameter over twenty micrometers. A few of small plate-like particles with length up to three micrometers also existed (Fig 2C), and their smooth planes and edges suggested that those particles were well-developed CCG-211790 crystals with oriented growth.

### Solubility

The solubility of CCG-211790 in water was 0.038 ±0.01 μg/mL, determined by HPLC analysis. CCG-211790 can be classified as practically insoluble compound (solubility less than 100 μg/mL) according to the definition of US Pharmacopeia [33]. Poor aqueous solubility of drugs is usually associated with pharmaceutical problems such as erratic absorption and poor bioavailability leading to insufficient drug concentrations at the site of action for pharmacological response [40, 41], and has become one of major challenges in pharmaceutical industries [42, 43].

*In vitro* dissolution testing is an important tool in pharmaceutics that can be used to predict *in vivo* performance of certain products or for the purpose of quality control [44, 45]. Prior to conducting a dissolution testing, the dissolution medium is usually selected based on the solubility data and the dose range of the drug product in order to achieve sink condition, where the volume of dissolution medium is at least three times the volume needed to form a saturated solution of the drug substance being measured [44]. SDS, synonymously sodium lauryl sulfate (SLS), is the most frequently used artificial surfactants in dissolution testing [46]. The presence of SDS in dissolution medium is able to increase the solubility of water-insoluble drugs and therefore, less quantity of dissolution medium is needed to meet sink conditions. Solubility of CCG-211790 in SDS solution after incubation at 37°C and 200 rpm for 24 or 48 h is shown in Table 1. To conduct a dissolution testing in 0.5% SDS solution at a dose of 5 mg of CCG-211790, a minimum of 500 mL of dissolution medium is required to meet sink condition.

**Table 1:** Solubility of CCG-211790 bulk powder in 0.5, 1.0 or 2.0% SDS solution after incubation in a shaking incubator at 37°C and 200 rpm for 24 or 48 h.

SDS concentration	Solubility (μg/mL)		
(*w/v*)	24 h	48 h	Mean
0.5%	32.9	26.8	29.9 ± 4.3
1.0%	68.5	63.8	66.1 ± 3.3
2.0%	161.4	149.7	155.5± 8.3

Solubility of CCG-211790 in various organic solvents was investigated using UV-Vis analysis, as seen in Table 2. Dimethylacetamide (DMA), dimethylformamide (DMF) and dimethyl sulfoxide (DMSO) have the highest solvent power for CCG-211790, which are 278.5, 215.4 and 66.7 mg/mL respectively. DMSO is a polar aprotic solvent that has been widely used in pharmaceutics and biological studies [47].

**Table 2:** CCG-211790 solubility in various organic solvents at room temperature.

Organic solvents	Solubility (mg/mL)
Acetonitrile	57.6
Ethanol	18.2
Glycerin	1.3
Isopropyl alcohol	15.4
Oleic acid	24.1
Dimethyl sulfoxide	66.7
Dimethylacetamide	278.5
Dimethylformamide	215.4
Canola oil	24.7
Coconut oil	37.2
Olive oil	24.3
Peanut oil	22.3
Safflower oil	22.7
Sesame oil	22.7
Sunflower oil	20.0

Higher solubility of DMSO for CCG-211790 over other solvents such as various edible oils and Acetonitrile indicates DMSO would be a promising organic solvent for CCG-211790 in preliminary studies. Edible oils listed in Table 2 are commonly used pharmaceutical excipients [47]. CCG-211790 solubility in these edible oil ranges from 20–37.2 mg/mL, giving potential for these edible oils to be used as vehicles for CCG-211790 in oral formulation development.

### Effect of pH on CCG-211790 stability

Solubility of CCG-211790 under various pH conditions in a mixture of acetonitrile/water (pH 1, 2 or 10) or acetonitrile/PBS (pH 3–9) when incubated at 37°C over a period of twelve weeks was shown in Table 3. While CCG-211790 degraded at pH 1, it maintained most chemical stability at pH 2–10, which includes most physiological conditions of between pH 5–7.

**Table 3:** Change of CCG-211790 concentration in the mixture solution of acetonitrile/water or acetonitrile/PBS with pH from 1–10 when incubated at 37°C over a period of twelve weeks.

pH	CCG-211790 concentration (μg/mL)			
	0 week	2 weeks	4 weeks	12 weeks
1	11.1	9.4*	7.2**	2.1**
2	11.6	11.9	11.2*	10.7*
3	11.9	11.9	11.4	11.7
4	10.9	11.4	11.6*	11.3
5	11.0	11.3*	11.5	11.2
6	11.1	11.4	11.0	10.1
7	11.8	11.2*	10.9*	11.5
8	11.2	11.2	11.1	11.6
9	11.0	11.3*	11.2	10.5
10	10.4	11.0*	10.5	10.7*

* Statistical significance compared with initial concentrations measured at 0 week when *P*<0.05.

** Statistical significance compared with initial concentrations measured at 0 week when *P*<0.01.

### Formulation of CCG-211790 for topical application

Because many MRSA infections are skin or soft tissue, a topical delivery of an anti-virulence agent would be therapeutically useful and would help test the proof of concept for controlling infections with an anti-virulence agent. Using Quality by Design principles, a topical formulation was developed composed of FDA approved “generally recognized as safe” (GRAS) excipients that are listed in the FDA’s Inactive Ingredient Database. In compliance with EP or USP the excipients contribute to the desired profile of the cream. The details of the topical compositions of CCG-211790 in the cream emulsion are listed in Table 4.

**Table 4:** Compositions of cream emulsions with different CCG-211790 drug loads.

Excipient	Component%			
	0% Drug load	0.3% Drug load	1% Drug load	3% Drug load
Isopropyl myristate (IPM)	10.5	10.5	10.5	10.5
Mineral oil	42.2	42.2	42.2	42.2
BRIJ^®^ 58	6.2	6.2	6.2	6.2
Stearyl alcohol	3.6	3.6	3.6	3.6
Cetyl alcohol	3.6	3.6	3.6	3.6
Xanthan gum	0.2	0.2	0.2	0.2
Water	33.4	33.1	32.4	30.4
Citric acid	0.07	0.07	0.07	0.07
Sodium citrate	0.23	0.23	0.23	0.23
CCG-211790	0	0.3	1	3
Total	100%	100%	100%	100%

The components included: mineral oil and isopropyl myristate (IPM) as oil phase, PEG 20 cetyl ether as surfactant, stearyl alcohol as stiffening agent, cetyl alcohol for emulsifier/stiffening, xanthan gum for thickening, water as aqueous phase and citric acid and sodium citrate as buffer. The oil phase solubilizes the active agent, CCG-211790, which is emulsified with water, surfactants, and thickening agents.

The drug release testing and performance parameters of the above-mentioned cream formulations were conducted using the standard Franz Chamber testing method for drug release and penetration and performance analysis. Drug release profile (Franz Chamber Assay) was carried out to measure drug release from this dosage form. For semisolid drug products (such as a cream formulations), release properties can be evaluated by *in vitro* release testing (IVRT). An *in vitro* release rate can reflect the combined effect of several physical and chemical parameters, such as solubility and particle size of the active pharmaceutical ingredient (API or drug) and rheological properties of the dosage form [48, 49]. The Franz diffusion cell can be used to assess drug release and skin permeability, leading to understanding of the relationships between skin, drug and formulation [48, 49]. Such testing is highly useful in the design and development of novel formulations, toxicity screening [50] and quality control of drug release profile if skin is used rather that artificial membranes [51–53]. The method of Ng et al. [54] with synthetic membranes was used, which has been demonstrated to correlate with human and pig skin for drug release profiling and penetration [55]. This method also allows for reproducible results compared to those using skin samples that vary from experiment to experiment. The drug release from topical formulation is different from other drug delivery systems. FDA has issued a guidance [56], in which section VII. claimed release was theoretically proportional to the square root of time (√t). Thus, the *in vitro* release study was performed as per the guidance. Some other studies also use the same way to characterize *in vitro* release [57, 58].

**Fig 3:**
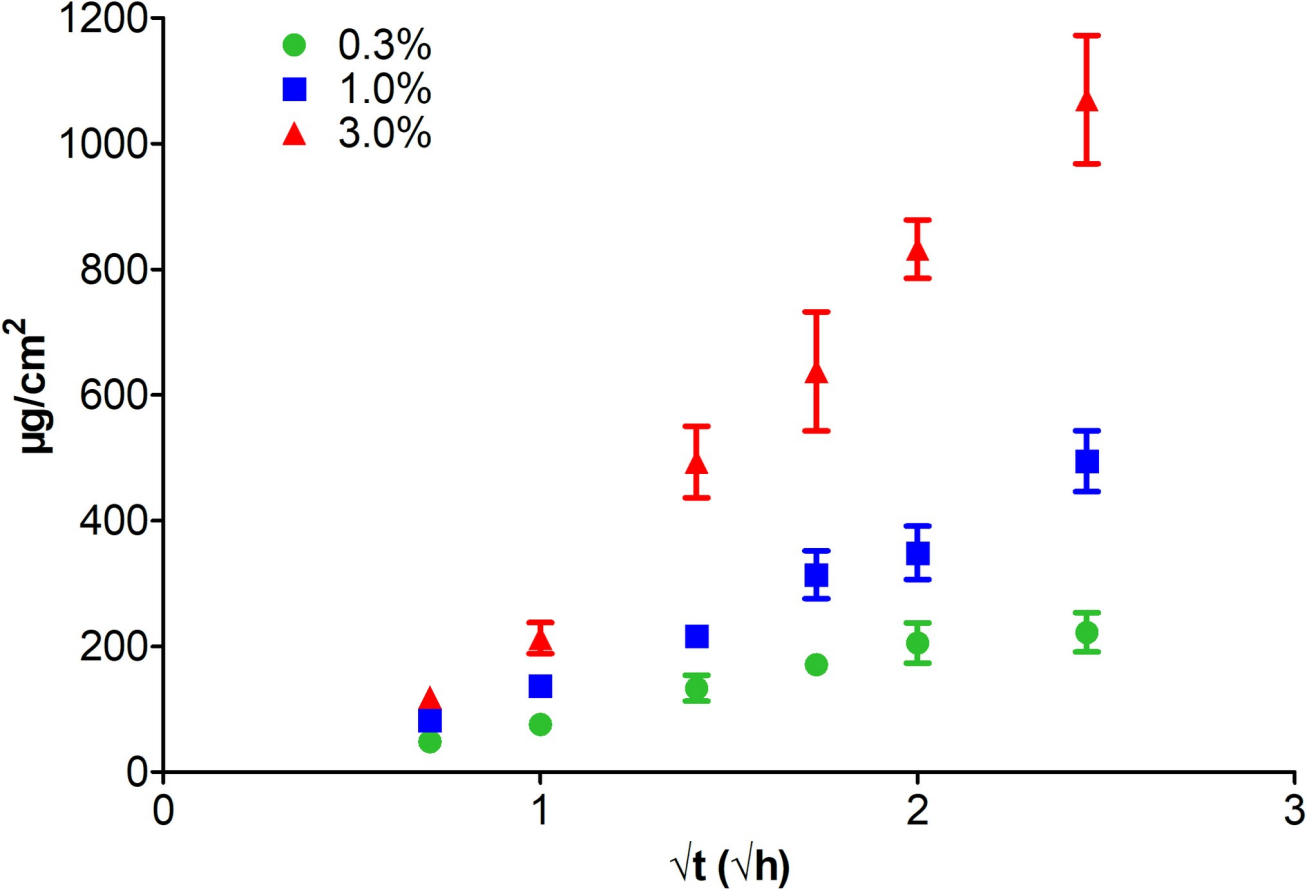
Drug release behavior (drug loading 0.3%, 1.0% and 3.0%).

The drug release parameters are included in Table 5. The drug release curves are illustrated in Fig 3. The concentration released over a 3h period for 0.3, 1% and 3%, was approximately 16 μg/mL (42 μM), 36 μg/mL (94 μM), and 77 μg/mL (200 μM), which is more than 30 times above the antibiofilm IC_50_.

**Table 5:** Drug release parameters for various cream formulations with CCG-211790.

Drug loading	Release Rate (μg/cm^2^/√h)	Cumulative Release Rate (%)	Cumulative Release (μg)	Final Concentration (μg/mL)	R^2^
0.3%	107±21	38±7	251±35	16±2	0.9398
1.0%	233±18	27±4	559±55	36±4	0.9637
3.0%	562±53.0	39±3	1209±116	77±7	0.9899

### Quality target product profile of the CCG-211790 cream

The quality target product profile was also tested and summarized in Table 6, whereas the stability data is included in Table 7.

**Table 6:** Quality target product profile.

QTPP Elements		Target	Justification
Dosage form		Cream	Match indication
Dosage design		O/W emulsion cream with CCG-211790 dissolved and dispersed in the cream base	
Route of administration		Topical	
Dosage strength		0.3%, 1.0% or 3.0% *w/w*	To be determined by animal test
Stability		No less than 24-month expiration dating period	Match the shelf life of similar products
Drug product quality attributes	Appearance	White smooth cream with dissolved and dispersed CCG-211790	Patient acceptability
	pH	5.0~5.4	No irritation
	Viscosity	12000 ~14000 cP	Needed for applicability and drug release control
	Identification	Positive for CCG-211790	Needed for clinical effectiveness
	Assay	90~110% of label claim	
	Content Uniformity	Top, middle and bottom of three containers. Nice assay values should be within 90.0%~110.0% label claim and RSD ≤ 5%.	Needed for clinical effectiveness
	Impurities	Any individual unknown: NMT 0.2%; total impurities: NMT 0.5%	Needed for safety
	Release behavior	Release rate within 10~30% in 6 h	To describe performance characteristics as a part of quality control procedure and rationalization for scale-up and post approval changes
	Preservatives content	Methyl paraben: 80.0~110.0% label claim;	Needed for ensuring antimicrobial effectiveness
	Microbial limits	Meet USP <61>	Needed for safety
	Residual solvents	Meet UPS <467>	Needed for safety
Container closure system		Container closure system qualified as suitable for this drug product	Needed to achieve the target shelf-life and to ensure stability during shipping and storage
Package integrity		No failure	Needed for stability, clinical effectiveness and safety

**Table 7:** Stability of cream formulation.

Time	Viscosity/cP		pH	
Day 1	13483±104		5.14	
Temperature	25°C	40°C	25°C	40°C
Day 14	17200±312	15167±1841	5.14	5.19
Month 1	13783±896	16600±656	5.33	5.06
Month 3	13170±442	18683±925	5.24	5.32
Month 6	12383±275	10667±493	5.35	5.19
Month 9	11817±448	8590±592	5.33	5.18
Deceased by	12%	36%		

## Discussion

In the ongoing SAR effort to develop the novel anti-virulence class of compounds [16, 17, 32, 38], 87 quinazolin-4-one derivatives were identified with > 20% inhibition of *S*. *aureus* biofilm at 100 or 50 μM [38]. Using the crystal violet (CV) staining microtiter-based biofilm assays, a large number of compounds were screened to identify potential anti-biofilm reagents. There have been many studies utilizing different assays to identify anti-biofilm agents [23]. Other commonly used biofilm assays include using Resazurin (REZ) and benzene sulfonic acid hydrate (XTT) assays that are based on the metabolic activity of the bacteria [23]. While there could be a discrepancy between the results of the different biofilm assays, CV staining screening is commonly used in many screening and testing studies for anti-biofilm reagents [59–61], which quantifies the biomass of the biofilm and is easier to standardize due to its simplicity and lower cost. Previous studies shown that the result of the CV staining of biofilm assay correlated well with slime analysis and quantification of bacteria genomic DNA [62]. Kong et al. have used an *in vivo Caenorhabditis elegans* (*C*. *elegans*)–*S*. *aureus* anti-infective screen to screen a collection of 35 benzimidazole derivatives to identify compounds with anti-biofilm activity with IC_50_ in the low μM range [61], in the similar range of IC_50_ as the anti-biofilm compound in this study. The *in vivo C*. *elegans*–*S*. *aureus* anti-infective screen identified compounds that can protect the nematodes from a lethal *S*. *aureus* infection. Since benzimidazole-derived molecules previously demonstrated anti-biofilm activity, CV staining assays were performed to identify benzimidazole compounds with anti-biofilm activity against *S*. *aureus*. The benzimidazole compound was able to disrupt biofilm formation. The bacteria appeared as a monolayer of dispersed cells scattered on the surface [61]. There are currently many efforts to develop anti-biofilm agents, which contain moieties such as imidazole, phenols, indole, triazole, sulfide, furanone, bromopyrrole, peptides that are capable of disrupt and disperse biofilm [63]. Many of the compounds are developed from scaffolds of naturally derived compounds [63]. Many of the compounds have reported IC_50_ in middle to high μM range, with some of compounds have reported IC_50_ in the low μM range [63], like in the current study.

The new compound CCG-211790 has better potency and a longer half-life when incubated with mouse liver microsomes than the previously published compounds CCG-203592 and CCG-205363 [17, 32]. This class of anti-biofilm compounds could inhibit the gene expression of a number of *S*. *aureus* virulence factors involved in biofilm formation [17]. The compound could disrupt the biofilm structure with less dense bacterial cell clusters in comparison with the well-formed biofilm structures treated with control vehicle [17]. It is interesting that in Kong et al’s. study, the benzimidazole compounds also disrupted the biofilm structure similarly. The benzimidazole derivatives also inhibited the expression of a number of virulence factors such as the clumping factors (Clf) and serine-aspartate repeat-containing proteins (Sdr) that promote adherence of bacteria to a substratum and induce staphylococcal biofilm formation [61]. Yet the gene expression profile affected by the quinazolin-4-one derivatives is different from the benzimidazole compounds. For example, it was observed that the quinazolin-4-one derivative markedly reduced expression of genes *spa*, *LrgA* and *psm*α [17], which weren’t affected by benzimidazole compound, suggesting the two classes of compounds could target different sets of virulence factor expression. Nevertheless, it suggests that there are multiple pathways that can be targeted to achieve anti-biofilm and anti-virulence activity.

It is thus concluded that this class of anti-biofilm compounds could disrupt biofilm formation and affect virulence gene expression in *S*. *aureus* [17]. On the other hand, the compounds will need to be tested for their *in vivo* efficacy at mitigating infection in animal models which is planned for future studies. CCG-211790 and its analogs represent a new class of anti-virulence compounds which can inhibit *S*. *aureus* virulence and biofilm formation and has the potential for treating biofilm-related diseases.

CCG-211790 is a novel anti-virulence compound with poor aqueous solubility (0.038 ±0.01 μg/mL) and can be regarded as an insoluble compound in water. CCG-211790 solubility in selected edible oils ranges from 20–37.2 mg/mL, giving potential for these edible oils to be used as vehicles for CCG-211790 in formulation development. DMSO has relatively high solvent power for CCG-211790 (66.7 mg/mL) and can be considered as a promising organic solvent for CCG-211790 in preliminary studies. CCG-211790 retains chemical stability at most physiological pH-values. Since poor aqueous solubility of drugs is usually associated with pharmaceutical problems, effort was thus made for the development of CCG-211790 formulations in order to enhance its pharmaceutical performance.

One important therapeutic application of this class of anti-biofilm compounds is to develop a topical formulation to treat SSTIs. The skin covers the whole body and is the largest organ in humans that could be subjected to various disorders and diseases. Topical products, which can be administered easily and are portable, are widely used in treating a variety of diseases. Semisolid topical preparations are most commonly used. By incorporating drugs into semisolids, they can exert their activity on the surface layers of tissues or with additional enhancers, penetrate into deeper layers at the site of action [64].

**Fig 4:**
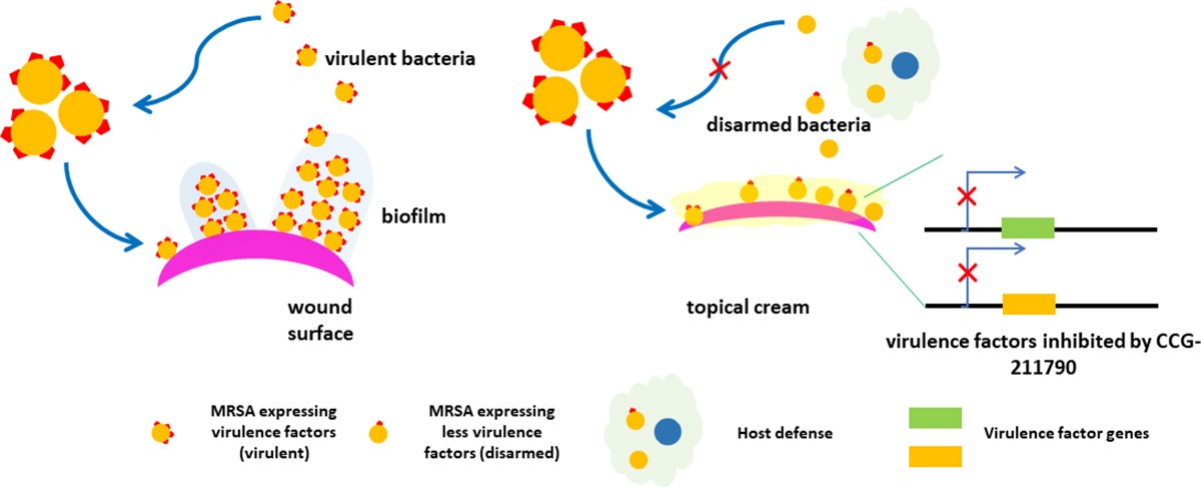
Topic formulation with CCG-211790 to treat wound infections via disarming the virulence factors of bacteria. Bacteria form biofilm in the wound, which is recalcitrant to host defense and antibiotics. Topical cream releases CCG-211790 to the site of infection to inhibit virulence factor expression and biofilm formation, to facilitate host defense to fight infections.

To explore the potential application of the novel anti-virulence compounds in treating SSTIs, a topical cream of CCG-211790 was then developed and evaluated to access the bacteria in a wound. The emulsion was developed to possess the acceptable properties for drug release, viscosity, pH, cosmetic elegance (odorless, non-greasy, spreadable, quick drying, and non-irritating) and stability with over nine months testing. This study thus demonstrated that topical formulation of this novel class anti-biofilm compounds can overcome the shortcomings of CCG-211790’s poor water solubility to enable it to be released to the infection sites (~33-156-fold of IC_50_) to exert its anti-biofilm activity against antibiotic resistant bacteria in the wound which is on the surface of the skin (Fig 4).

The compound has some cytotoxicity at high concentration (50 μM). Stratum corneum will greatly diminish the damage to the host skin cells by any potential cytotoxicity effect of the compound. Further studies will be needed to assess the toxicity and tolerability of this topical formulation along with efficacy studies in skin infection models.

Topical drug delivery could provide high local concentration of anti-virulence agents and be used to validate the mechanism of action in animal studies and clinical trials to demonstrate the potential of novel anti-virulence agents.

## Conclusion

In summary, a novel anti-virulence compound was developed that could potentially be used to treat or prevent SSTIs caused by MRSA. The mechanism of action will be to prevent or reduce biofilm formation by antibiotic resistant pathogens and therefore help reduce or eliminate bacterial skin infections. The anti-virulence compound is different from conventional antibiotics by not directly killing bacteria or blocking their proliferation, inducing less selective pressure for drug resistance. The mechanism of action is “disarming” bacteria by lowering their ability to form biofilm and harm the host. Blocking virulence factors that allow bacterial adaptation to environmental changes will make bacteria more vulnerable to host defense and immunity [8, 65]. A topical formulation with the acceptable properties for drug release, viscosity, pH, cosmetic elegance and stability has been developed to overcome the poor water solubility of the compound to enhance its therapeutic potential for treatment of SSTIs, in which antibiotic resistance and biofilm formation are major challenge for conventional treatments.

## Supporting information

S1 Table Original results of the effect of CCG-211790 on the biofilm formation of NRS384. (DOCX)

S2 Table Original results of the effect of CCG-211790 on the mammalian cell viability. (DOCX)

S3 Table Original results of drug release behavior (drug loading 0.3%, 1.0% and 3.0%). (DOCX)
