# Supplementary material for: Physicochemical properties and formulation development of a novel compound inhibiting Staphylococcus aureus biofilm formation
Source: PLoS One. 2021 Feb 8;16(2):e0246408. doi: 10.1371/journal.pone.0246408 (PMC7870075; doi:10.1371/journal.pone.0246408)
Supplement: S1 Table — (DOCX) [file pone.0246408.s001.docx]

**S1 Table.** Original results of the effect of CCG-211790 on the biofilm formation of NRS384

| CCG-211790 concentration (µM) | Inhibition of biofilm formation (vs treated with DMSO %) | | | | | | | | |
| --- | --- | --- | --- | --- | --- | --- | --- | --- | --- |
| 0.39 | 3.94 | -13.46 | 12.53 | 16.63 | 8.53 | 4.38 | 18.40 | 26.22 | 15.26 |
| 0.78 | 8.82 | 2.09 | 10.90 | 24.07 | 16.41 | 12.91 | 26.42 | 26.03 | 21.14 |
| 1.56 | 29.00 | 47.10 | 40.37 | 45.73 | 49.67 | 47.05 | 51.47 | 53.42 | 52.45 |
| 3.13 | 41.76 | 55.22 | 48.72 | 60.18 | 56.46 | 59.74 | 54.01 | 55.58 | 57.14 |
| 6.25 | 62.41 | 64.27 | 61.48 | 70.02 | 63.24 | 70.24 | 69.08 | 70.25 | 66.93 |
| 12.50 | 65.20 | 63.57 | 64.97 | 75.93 | 70.24 | 70.02 | 75.73 | 75.34 | 73.19 |
| 25.00 | 70.30 | 69.84 | 74.25 | 79.43 | 78.99 | 75.05 | 76.52 | 81.02 | 80.63 |
| 50.00 | 79.58 | 79.12 | 79.35 | 82.49 | 82.28 | 82.28 | 80.23 | 79.06 | 83.76 |
