# Supplementary material for: Physicochemical properties and formulation development of a novel compound inhibiting Staphylococcus aureus biofilm formation
Source: PLoS One. 2021 Feb 8;16(2):e0246408. doi: 10.1371/journal.pone.0246408 (PMC7870075; doi:10.1371/journal.pone.0246408)
Supplement: S2 Table — (DOCX) [file pone.0246408.s002.docx]

**S2 Table.** Original results of the effect of CCG-211790 on the mammalian cell viability

| CCG-211790 concentration (µM) | 0 | 6.25 | 12.5 | 25 | 50 |
| --- | --- | --- | --- | --- | --- |
| Cell viability % | 102.78 | 79.57 | 80.79 | 68.95 | 61.27 |
|  | 97.22 | 86.99 | 82.11 | 69.05 | 57.46 |
|  | 111.19 | 84.77 | 70.18 | 56.68 | 48.66 |
|  | 88.81 | 85.92 | 71.70 | 66.50 | 56.82 |
| Mean of cell viability | 100.00 | 84.31 | 76.19 | 65.29 | 56.05 |
| Standard deviation of cell viability | 9.42 | 3.29 | 6.12 | 5.86 | 5.30 |
