# Supplementary material for: Physicochemical properties and formulation development of a novel compound inhibiting Staphylococcus aureus biofilm formation
Source: PLoS One. 2021 Feb 8;16(2):e0246408. doi: 10.1371/journal.pone.0246408 (PMC7870075; doi:10.1371/journal.pone.0246408)
Supplement: S3 Table — (DOCX) [file pone.0246408.s003.docx]

**S3 Table.** Original results of drug release behavior (drug loading 0.3%, 1.0% and 3.0%)

| √t (√h) | 0.3% | | | 1.0% | | | 3.0% | | |
| --- | --- | --- | --- | --- | --- | --- | --- | --- | --- |
| 0.71 | 47.26 | 55.02 | 43.74 | 99.01 | 67.64 | 80.56 | 104.17 | 127.11 | 126.8 |
| 1.00 | 77.79 | 86.76 | 63.28 | 140.97 | 124.75 | 145.88 | 223.51 | 231.15 | 185.47 |
| 1.41 | 149.33 | 141.12 | 110.14 | 203.9 | 216.52 | 227.99 | 529.77 | 523.31 | 427.85 |
| 1.73 | 165.31 | 184.79 | 163.11 | 298.54 | 286.4 | 357.55 | 702.73 | 681.52 | 528.88 |
| 2.00 | 242.24 | 186.53 | 187.58 | 385.06 | 302.09 | 359.34 | 829.41 | 880.2 | 786.99 |
| 2.45 | 251.28 | 189.13 | 226.96 | 535.4 | 508.45 | 441.23 | 1115.27 | 1141.93 | 953.07 |
